# Crystal structure of human IPS-1/MAVS/VISA/Cardif caspase activation recruitment domain

**DOI:** 10.1186/1472-6807-8-11

**Published:** 2008-02-28

**Authors:** Jane A Potter, Richard E Randall, Garry L Taylor

**Affiliations:** 1 Centre for Biomolecular Sciences, University of St Andrews, St Andrews, Fife, KY16 9ST, UK

## Abstract

**Background:**

IPS-1/MAVS/VISA/Cardif is an adaptor protein that plays a crucial role in the induction of interferons in response to viral infection. In the initial stage of the intracellular antiviral response two RNA helicases, retinoic acid inducible gene-I (RIG-I) and melanoma differentiation-association gene 5 (MDA5), are independently able to bind viral RNA in the cytoplasm. The 62 kDa protein IPS-1/MAVS/VISA/Cardif contains an N-terminal caspase activation and recruitment (CARD) domain that associates with the CARD regions of RIG-I and MDA5, ultimately leading to the induction of type I interferons. As a first step towards understanding the molecular basis of this important adaptor protein we have undertaken structural studies of the IPS-1 MAVS/VISA/Cardif CARD region.

**Results:**

The crystal structure of human IPS-1/MAVS/VISA/Cardif CARD has been determined to 2.1Å resolution. The protein was expressed and crystallized as a maltose-binding protein (MBP) fusion protein. The MBP and IPS-1 components each form a distinct domain within the structure. IPS-1/MAVS/VISA/Cardif CARD adopts a characteristic six-helix bundle with a Greek-key topology and, in common with a number of other known CARD structures, contains two major polar surfaces on opposite sides of the molecule. One face has a surface-exposed, disordered tryptophan residue that may explain the poor solubility of untagged expression constructs.

**Conclusion:**

The IPS-1/MAVS/VISA/Cardif CARD domain adopts the classic CARD fold with an asymmetric surface charge distribution that is typical of CARD domains involved in homotypic protein-protein interactions. The location of the two polar areas on IPS-1/MAVS/VISA/Cardif CARD suggest possible types of associations that this domain makes with the two CARD domains of MDA5 or RIG-I. The N-terminal CARD domains of RIG-I and MDA5 share greatest sequence similarity with IPS-1/MAVS/VISA/Cardif CARD and this has allowed modelling of their structures. These models show a very different charge profile for the equivalent surfaces compared to IPS-1/MAVS/VISA/Cardif CARD.

## Background

In the cells of higher eukaryotes, recognition of virally-derived RNA intermediates produced during replication activates signalling cascades that trigger the production of interferons (IFNs) and other cytokines, which in turn mediate innate immunity and modulate subsequent adaptive immunity [1,2]. The innate immune system utilizes pattern-recognition receptors (PRRs) to detect conserved molecular patterns on certain types of molecule that are not produced by the host but are characterisitic of invading microorganisms [3]. There are two categories of PRRs involved in the induction of type I IFNs: Toll-like receptor (TLRs) and RIG-I-like receptors (RLRs). The transmembrane-anchored TLR family members, present on cell surfaces or in endosomes, bind extracellular viral components [4]. In contrast, the initial detection of intracellular viral nucleic acids occurs via a TLR-independent pathway in which two RNA helicases, retinoic acid inducible gene-I (RIG-I) and melanoma differentiation-association gene 5 (MDA5), are able to sense viral RNA in the cytoplasm [5,6].

RIG-I and MDA5 are functionally-related cytosolic proteins that each contain two N-terminal caspase activation and recruitment (CARD) domains and a DExD/H-box RNA helicase domain. These RLRs are activated by different types of RNA molecules produced as by-products of virus replication, such as double stranded RNA or uncapped RNA bearing 5' triphosphates, which are not found in uninfected cells [7-9]. The C-termini of RIG-I and MDA5 function as regulatory repressor domains, deletion of which results in constitutive signalling to the IFN-β promoter [10]. Upon binding of dsRNA (or other ligands) to the helicase domain, RIG-I and MDA5 are presumed to undergo structural alteration and multimerization, thereby unmasking the CARDs and enabling them to recruit downstream signal transducer proteins. The tandem N-terminal CARD domains of RIG-I or MDA5 are able to act as dominant activators: overexpression of either of these tandem domains results in the induction of IFN production without viral infection [5,6,11]. RIG-I and MDA5 signalling results in the activation of IKKε and TBK-1, two serine/threonine kinases that phosphorylate IRF3 and IRF7 [12-14]. Upon phosphorylation, IRF3 and IRF7 translocate to the nucleus and subsequently induce IFN-α and IFN-β gene transcription [15].

The adaptor protein that acts as an intermediate between RIG-I/MDA5 detection of viral RNA and downstream activation events was discovered by four groups in 2005 and given four different names: IPS-1 (IFN-β promoter stimulator protein 1) [16], MAVS (mitochondrial antiviral signalling protein) [17], VISA (virus-induced signalling adaptor) [18] and Cardif (CARD adaptor inducing IFN-β) [19]. IPS-1 (as it will be referred to from hereon) is a 62 kDa protein containing an N-terminal CARD domain, a proline-rich region and a transmembrane domain that targets it to the outer mitochondrial membrane [17]. Overexpression of IPS-1 activates the IFN-α, IFN-β and NF-κB promoters, requiring interactions of the kinases TBK1 and IKKε with IPS-1 for the activation of these promoters [16,20]. The proline rich region interacts with a number of signalling molecules including TRAF6, TRAF2 [18], RIP1, FADD [16] and TRAF3 [21], suggesting that IPS-1 plays a role in TLR3-mediated pathways in addition to TLR-independent, RIG-I/MDA5-mediated signalling [18]. It has also been demonstrated that both the IPS-1 CARD and C-terminal mitochondrial membrane-targeting domains of IPS-1 are essential for IFN-β induction [17]. IPS-1 has been identified as a target for the NS3/4A protease of hepatitis C virus (HCV), which cleaves IPS-1 from the mitochondrial membrane and abolishes its ability to signal to the IFN-β promoter [19].

The CARD domain of IPS-1 interacts with the N-terminal regions of RIG-I or MDA5 most probably through homotypic CARD-CARD associations [16], and both CARD domains of RIG-I are required for the interaction with IPS-1 CARD [18]. In the case of MDA5, it has been shown that dihydroxyacetone kinase (DAK) binds to the CARD domains of MDA5, suggesting that DAK acts as a negative regulator of MDA5 that is released upon a conformational change induced by viral RNA binding, allowing the MDA5 CARD domains to bind to IPS-1 CARD [22]. In the case of RIG-I, it has been shown that the second CARD region can be ubiquitinated, and that the level of ubiquitination correlates with the signal transduction activity of RIG-I, and may facilitate its interaction with IPS-1 [23]. Other proteins are known to bind to IPS-1 CARD to regulate signalling, as it has been shown that the Atg5-Atg12 conjugate, a key regulator of the autophagic process, interacts with IPS-1 CARD and the CARD domains of RIG-I thereby blocking interferon production [24].

CARDs are members of the death domain (DD) superfamily, which also includes the death domain, death effector domain and pyrin domain subfamilies [25]. Members of the DD superfamily play a critical role in the assembly of oligomeric signalling complexes in apoptotic and inflammatory processes. In the intrinsic apoptosis pathway for example, release of cytochrome *c *from the mitochondria into the cytoplasm induces Apaf-1 to recruit caspase-9 via a CARD-CARD interaction [26]. As a first step towards understanding the structural basis of the mode of action of IPS-1, we report the crystal structure of the human IPS-1 CARD domain to a resolution of 2.1Å. The IPS-1 CARD domain shares homology with the first CARD domains of MDA5 and RIG-I (25% and 20% sequence identity respectively), which allows homology modelling of these related CARDs that, together with the second CARD domains, are known to interact with IPS-1 CARD.

## Results and Discussion

### Structure overview

The crystal structure of an engineered chimera (MBP-CARD), in which human IPS-1 CARD is fused to the C-terminus of MBP, was solved to 2.1Å resolution. IPS-1 CARD was crystallized as an MBP fusion construct as untagged IPS-1 CARD exhibited poor solubility and stability. Crystallization required a short linker region between MBP and IPS-1 CARD, as has been successful in a number of other chimeric fusion protein structures, for example an MBP-homeobox domain chimera (pdb code 1mh4) [27], an MBP-Argonaute2 PAZ domain chimera (1r6z) [28] and an MBP-Nedd8-activating enzyme E1 subunit chimera (2nvu) [29].

**Figure 1:**
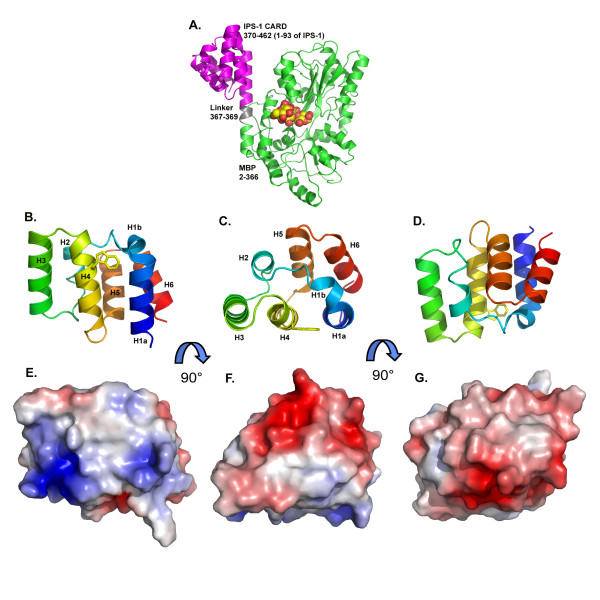
**Structure of the human IPS-1 CARD domain.** (A) Structure of the MBP-CARD fusion structure with maltotetraose bound in the MBP binding site. (B,C,D) orthogonal views of the IPS-1 CARD domain colored from blue at the N-terminus to red at the C-terminus with Trp56 highlighted. (E,F,G) surface representations colored by electrostatic potential in the same orientations as the images above.

The asymmetric unit contains one molecule of MBP-CARD comprising residues 2–366 of MBP, a 3 amino acid linker (sequence NSA), and residues 1 to 93 of human IPS-1, but with a mutation from Pro to Ala at position 2 introduced during the cloning. The MBP and IPS-1 CARD components of the fusion protein form two distinct domains (Fig. 1A). The 3 amino acid linker forms a helix that connects the final helix of MBP to the first helix of IPS-1 CARD.

IPS1-CARD exhibits the characteristic topology of the DD superfamily, comprising six tightly packed α-helices arranged in a Greek-key motif (Fig. 1B,C,D). Helix 1, often severely kinked in CARD structures, is divided into two smaller helices: H1a, from residues 4 to 14 and H1b, which is a short 3_10 _helix encompassing residues 16 to 19. Helix 2 extends from residues 24 to 30, the last three residues of which also form a 3_10 _helix. The remaining four helices comprise residues 36 to 49 (H3), residues 51 to 64 (H4), residues 66 to 77 (H5) and residues 80 to 90 (H6). The loops between the helices are well defined.

### Comparison with other CARD structures

Several other CARD structures have been determined to date: NMR structures are available for RAIDD CARD [30], NOD1 CARD [31] and ICEBERG CARD [32]. Crystal structures of CED-4 CARD [33], NOD1 CARD [34] Apaf-1 CARD [35] and a complex of Apaf-1 CARD with procaspase-9 CARD [36] have been solved. All of these CARDs belong to proteins involved in apoptotic pathways.

**Figure 2:**
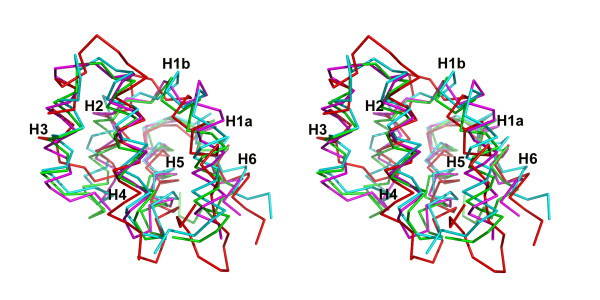
**Stereo diagram of superposed CARD domains.** IPS-1 CARD (magenta), Apaf-1 CARD (green), the prodomain of procaspase-9 CARD (cyan) and chain K of the oligomeric death domain complex (red).

The superposition of representative CARD structures with IPS-1 CARD is shown in Fig. 2. The closest structural homologues are Apaf-1 (pdb code 2 ygs, rmsd of 1.79Å for 81 Cα atoms), the prodomain of procaspase-9 (3 ygs, rmsd of 2.38Å for 85 Cα atoms) and several monomers of the oligomeric death domain complex (2 of 5, ~2.5Å for ~80 Cα atoms). There are two main differences in secondary structure that separate IPS-1 CARD from these CARDs. Helix 2 in IPS-1 CARD is truncated relative to the other known CARDs, a feature which is accompanied by a shortening of the loop interconnecting H2 and H3. By contrast, H3 is about 6 residues longer in IPS-1 CARD relative to the other CARD structures with H3 and H4 connected by only a single glycine residue.

### Surface characteristics

The surface of human IPS-1 contains two highly charged patches (Fig. 1E,F,G). Helices H1a, H3 and H4 form a flat surface that is largely positively charged due to Lys7, Lys10, Arg14, Arg37, Arg41, His57, Arg64 and Arg65. On the opposite side of the molecule, a negatively charged patch covers the surfaces helices H2 and H6 and their preceding loops. Residues contributing to this acidic region are Asp23, Glu26, Glu80, Asp83, Asp86 and Glu87.

**Figure 3:**
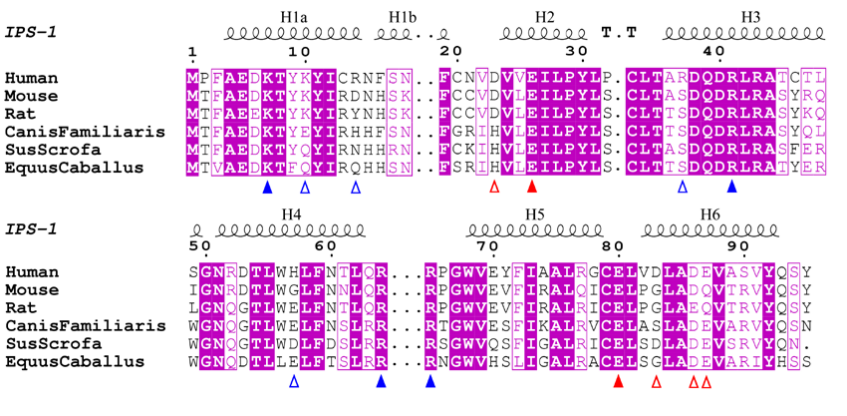
**Sequence alignment of IPS-1 CARDs from mammalian species.** Blue triangles indicate basic residues contributing to positive surface regions. Red triangles indicate residues contributing to the negative patch (open triangles are non-conserved, closed triangles are conserved).

The sequence alignment of IPS-1 CARDs from a number of species is shown in Fig. 3. The positions of residues contributing to the charged patches are indicated. In the negatively charged patch, residues Glu26 and Glu80 are conserved, Asp86 is always an Asp or Glu, Glu87 is always a Glu or Gln, whereas Asp23 and Asp83 are not conserved. Residues Lys7, Arg41, Arg64 and Arg65, present in the positively charged patch, are conserved across the IPS-1 family, and although Arg14 is not conserved, the preceding residue is an arginine in all other species.

Interestingly, the structure possesses a largely conserved surface tryptophan residue, Trp56 (leucine in horse) that interrupts the positive patch. This residue is disordered in the human IPS-1 CARD structure, the major conformer (60% occupancy) has its whole face exposed to solvent and stacks against Phe16, which is either Phe or His in other IPS-1 CARDs. In the other conformer (40% occupancy) the tryptophan side chain is fully exposed which in turn exposes Phe16 face-on to solvent. Also contributing to this hydrophobic patch is Tyr9, largely conserved across the IPS-1 CARDs, except for horse where it is a phenylalanine.

### Modelling MDA5/RIG-I CARD domains

**Figure 4:**
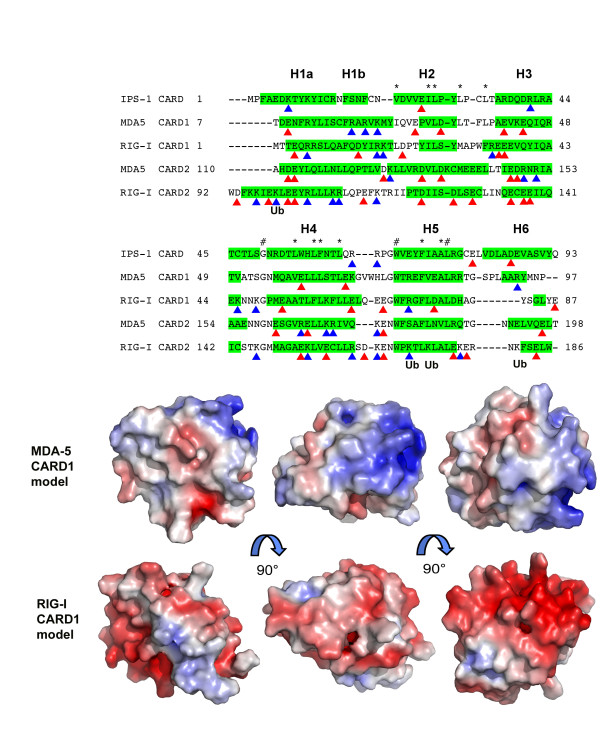
**Sequence alignment of human IPS-1 CARD domain with human MDA5 and human RIG-I CARD domains.** The experimentally determined helices of IPS-1 CARD, and the predicted helical regions of the other CARDs are highlighted in green. Conserved hydrophobic core residues are indicated by *, and totally conserved residues by #. The blue and red triangles indicate the positive and negative residues, respectively, that are conserved across all species for the respective CARD domain. Lysines of RIG-I that can be ubiquitinated by TRIM25 E3 ubiquitin ligase are labelled 'Ub'. Below are three orthogonal views of homology models of the first CARD domains of MDA5 and RIG-I, colored by electrostatic surface potential. The views are equivalent for those shown for IPS-1 CARD in Figure 1.

The annotations in the protein sequence databases relating to the boundaries of the CARD domains are not always an accurate reflection of the true boundaries. For example, the IPS-1 CARD domain is indicated as running from residue 10 to 77, whereas the crystal structure clearly shows that it runs from 1 to 93. Using the consensus secondary structure prediction within PHYRE [37], the first and second CARD domains of human MDA5 are likely to encompass residues 7 to 97 and 110 to 198, respectively, assuming a similar six-helical bundle topology. Similarly, the two human RIG-I CARD domains are likely to span residues 1 to 87 and 92 to 186. The first CARD domains of human MDA5 (MDA5CARD1) and RIG-I (RIGICARD1) share greater amino sequence identity, 25% and 22% respectively, to IPS-1 CARD than to any other CARD domain of known structure, for example MDA5CARD1 shares 15% identity with Apaf-1 CARD. Several hydrophobic amino acids are conserved across the IPS-1, RIG-I and MDA5 CARD domains (marked with a * or # in Fig. 4), and form part of the hydrophobic core of the IPS-1 CARD structure, which puts constraints on the relative orientations of the helices H2, H4 and H5. Models of MDA5CARD1 and RIGICARD1 were therefore constructed using homology modelling. Relative to IPS-1 CARD, MDA5CARD1 has two extra residues in the loop connecting H1b and H2, and three extra residues, including a tryptophan, in the loop between H4 and H5 (Fig. 4). It was assumed that MDA5CARD1 contained a kink between H1a and H1b as seen in IPS-1 CARD and all other CARD structures, even though secondary structure prediction suggested a continuous helix. RIGICARD1 is predicted to be similar to MDA5CARD1 except for a longer H3, a shorter H4–H5 loop and a much shorter H6.

The MDA5CARD1 model suggests that H1b is a 6 or 7-residue long α-helix, in contrast to the four amino acid 3_10 _helix seen in IPS-1 CARD. The surface of the MDA5CARD1 model shows that this helix is highly positively charged, containing residues Arg19, Arg21 and Lys23 (conserved across all species). This together with Arg84 and Arg85 at the end of H5 and Arg93 on H6 (also conserved across all MDA5s), create a highly-positively charged region on the surface of MDA5CARD1 (Fig. 4). The remainder of the molecule carries a mixed charged profile with a mainly overall negative charge due to acidic residues conserved across all species at positions 9, 29, 33, 41, 44, 60 and 67. The surface views of MDA5CARD1 in Fig. 4 are in the same orientations as the IPS-1 CARD in Fig. 1 and illustrate the very different charge profiles of the two molecules. The RIGICARD1 model suggests a very different charge profile to MDA5CARD1 (Fig. 4), in particular the surface is predominantly negatively-charged in the regions of H3 due to residues Glu34 and Glu35, conserved across all RIG-Is, and H4, H5 and H6 due to residues Glu63, Glu67, Asp 75 and Glu 87, conserved across all RIG-Is.

The sequence identity between the first CARD domains of human MDA5 and human RIG-I is 23%, and between the second CARD domains is 20%. The identity between the two CARD domains of MDA5 is 22% and between the two RIG-I CARD domains is 19%. As shown in Fig. 4, there is no obvious conservation of charged residues between the helicase CARD domains. Helices 3, 4 and 5 are predicted to be of similar length, with a conserved glycine between H4 and H5, but the extent of H1, H2 and H6 appear highly variable. The variability of the second CARD domains of MDA5/RIG-I precluded their modelling in this study. It has been shown that the second CARD domain of RIG-I can be ubiquitinated at several lysines (indicated in Figure 4), and that in particular ubiquitination at Lys172 in human RIG-I may facilitate its interaction with IPS-1 CARD [23], although Lys172 is not conserved across species, and is a glutamine in rat and mouse RIG-I.

### CARD-CARD associations

The crystal structure of the caspase-recruitment domain of Apaf-1 in complex with the prodomain of procaspase-9 is the only CARD-CARD complex structure determined to date [36]. Complex formation is mediated by electrostatic interactions between a negatively-charged convex region on Apaf-1 CARD (involving helices H2 and H3) and a positively-charged concave surface on procaspase-9 (involving helices H1a, H1b and H4), which are reinforced by van der Waals and H-bond interactions. The oligomeric PIDDosome complex revealed an assembly of 12 death domains that form a set of DD interactions classified into three types [38,39]. A type I interaction involves residues in H1 and H4 of the first DD interacting with residues of H2 and H3 of the second DD, with both polar and hydrophobic interactions at the interface. The Apaf-1 CARD:procaspase-9 CARD interaction is an example of a type I association. A type II interaction involves H4 and the H4–H5 loop of the first DD interacting with the H5–H6 loop and H6 helix of the second DD, with the interaction mainly involving charged residues. A type III interaction involves residues in H3 of the first DD interacting with residues near the H1–H2 and H3–H4 loops of the second DD, with the interaction being a mixture of polar, charged and hydrophobic [39].

Assuming that the CARD-CARD associations of IPS-1 with MDA5/RIG-I conform to one of the three types described above, then the basic face of IPS-1 CARD with the hydrophobic patch involving Trp56 and Tyr9 may associate via a type I interaction with the H2 and H3 regions of one of the CARD domains of the helicases. Similarly, the negatively-charged region of IPS-1 CARD involving H6 and its preceding loop may associate via a type II interaction with the H4 and H5 regions of the other helicase CARD domain.

## Conclusion

The crystal structure of the CARD domain of IPS-1 has been determined to 2.1Å resolution as part of a fusion construct with maltose-binding protein that allowed solubilization and crystallization. The structure is that of a typical member of the death domain superfamily, comprising a six-helix bundle with a Greek-key motif topology. The surface charge distribution, conserved across IPS-1 from a variety of mammalian species, shows an asymmetric distribution typical of CARD domains with positive and negative areas on opposite sides of the protein that are probably involved in electrostatically-driven homotypic CARD-CARD interactions. One unusual feature that probably explains the lack of solubility of non-tagged IPS-1 CARD is a disordered tryptophan (Trp56) that exposes its whole face in the centre of the flat, positively-charged surface of the protein involving helices H1, H3 and H4. This tryptophan is conserved in most species, suggesting that one CARD-CARD association made by IPS-1 involves a Type I interaction with helices H2 and H3 of one of the helicase CARD domains.

Homology modelling of the first CARD domains of MDA5 and RIG-I based on the structure of IPS-1 CARD suggests that they have a completely different surface charge profile compared to IPS-1 CARD, and in comparison with each other. Alignment of the CARD domains of MDA5 and RIG-I, together with secondary structure prediction and location of conserved residues suggests that although the dual CARD domains of RIG-I and MDA5 interact with the IPS-1 CARD domain, they may do so in quite different ways that may, of course, also involve the peptide linking the two CARD domains. In the case of RIG-I, the interaction may also involve ubiquitin at Lys172 in some species. This apparent variability in CARD-CARD interactions between the helicases and IPS-1 suggests a differential signalling mechanism for the two helicases. A detailed understanding of this recognition will only come with the elucidation of the complex of IPS-1 CARD with the CARD domains of both helicases.

## Methods

### Cloning and expression

The human IPS-1 CARD gene fragment was amplified from vector DNA by PCR. Primers were designed to incorporate an *Nco*I site at the 5' site and an *EcoR*I site following the termination codon. The fragment was cloned initially into a modified MBP-fusion vector with an N-terminal six-histidine tag and a TEV cleavage site upstream of the initiation codon of the IPS-1 gene. The *Nco*I site introduced a mutation at residue 2 in the IPS-1 CARD sequence from proline to alanine. The expressed fusion product therefore comprised N-terminally His-tagged MBP, a 21-amino acid linker including a TEV cleavage site and residues 1 to 93 of IPS1. Attempts to crystallize the protein either after cleavage from MBP or as a fusion with MBP were unsuccessful. A modified form of the MBP-IPS-1 CARD construct was therefore produced using reported methods [40], in which the flexible 21 residue linker containing the TEV cleavage site between MBP and IPS-1 CARD was reduced to just 3 amino acids.

The fusion protein was expressed in *E. coli *Rosetta (DE3)cells (Novagen). An overnight culture was used to inoculate 1L of Luria-Bertani medium supplemented with ampicillin. Cells were grown at 37°C to an OD_600 _of 0.6, at which point a final concentration of 0.4 mM isopropyl-β-D-thiogalactopyranoside (IPTG) was added to induce expression of the recombinant fusion product. After a further 16 h growth at 20°C cells were harvested by centrifugation.

### Purification and crystallization

Cell pellets were resuspended and sonicated in 20 mM TrisHCl pH8.5, 100 mM NaCl, 5 μg ml^-1 ^DNaseI, EDTA-free protease inhibitor cocktail (Roche). MBP-CARD was purified from clarified cell lysate by nickel column and amylose column purification using standard protocols. Pure protein was dialysed into 20 mM TrisHCl pH 8.5, 100 mM NaCl, 10 mM maltose and concentrated to 10 mg ml^-1 ^for crystal trials.

Sitting drop vapour diffusion crystal trials were carried out at 293 K using a nano-drop crystallization robot (Cartesian HoneyBee, Genomic Solutions) as part of the Hamilton-Thermo Rhombix system. An initial hit was obtained in condition 90 (2.2 M ammonium sulphate, 20% glycerol) of the NeXtal Ammonium Sulphate screen (Qiagen). Optimised crystals, which took approximately two weeks to appear, were grown in drops containing 1 μl of 12 mg ml^-1 ^protein and 1 μl of reservoir solution (2 M ammonium sulphate, 24% (v/v) glycerol).

### X-ray data collection and refinement

Crystals, which already contained sufficient glycerol for cryoprotection, were flash frozen in a nitrogen stream. A 2.1Å dataset was collected on beam-line ID14-2, ESRF, Grenoble. Crystals belonged to space group P4_1/3_2_1_2 with unit cell dimensions a = b = 99.3Å, c = 163.2Å, α = β = γ = 90°. The data were processed using MOSFLM [41] and scaled with the program SCALA in the CCP4 suite [42]. The structure was determined by molecular replacement with the program PHASER [43] using the known structure of MBP as phasing model (PDB accession number 1anf [44]). One molecule of MBP was found in the asymmetric unit for space group P4_1_2_1_2 and the remaining IPS-1 CARD portion of the structure was traced by ARP/wARP [45]. Refinement in Refmac5 [46] and model building using COOT [47] yielded a model with an R_cryst _of 0.18 and an R_free _of 0.22. During refinement, difference electron density in the oligosaccharide binding site of MBP clearly indicated the presence of four glycopyranoside rings and was therefore modelled as maltotetraose, the presence of which may be attributable to impurities in the maltose used to elute the protein from the amylose column. The mode of binding of maltotetraose to MBP is similar to that seen in previous structures [44,48]. Five electron density peaks, which were significantly higher than those of water molecules and coordinated to at least one basic residue, were assigned as sulphate ions as the crystals were grown in 2 M ammonium sulphate. TLS refinement [49] was performed in Refmac5 with 10 TLS groups, as defined by the TLSMD server [50]. Data collection and refinement statistics are given in Table 1.

**Table 1:** X-ray data collection and refinement statistics. Numbers in parentheses refer to the highest resolution shell.

Space group	P4_1_2_1_2
Unit cell edges (Å)	a = b = 99.3, c = 163.2
X-ray source, wavelength (Å)	ID14-2, 0.934
Resolution range	32.6-2.1Å
No. of unique observations	48, 262
Completeness (%)	99.8 (99.9)
Redundancy	7.1 (7.2)
R_merge_	0.072 (0.364)
<I/σI>	16.1 (4.4)
*Refinement*	
No. of reflections work/test	45,853/2,404
No. of protein atoms	3,665
No. of ligand atoms	45
No. of waters	202
Average B-factors (Å ^2^) protein/waters	21/34
R_cryst_	0.181
R_free_	0.220
r.m.s.d. bond distance (Å)	0.017
r.m.s.d bond angle (°)	1.5

*R*_merge _= ∑_*hkl *_∑_*i*_|*I*_*hkl*,*i *_- ⟨*I*_*hkl*_⟩|∑_*hkl*_⟨*I*_*hkl*_⟩ R_cryst _and R_free _= (Σ||F_o_| - |F_c_||)/(Σ|F_o_|)

Model quality was assessed by both PROCHECK [51] and MolProbity [52]. Ramachandran statistics indicate that 93.5% of the residues are located in the most favoured regions of the plot with the remaining 6.5% present in additional allowed regions. Coordinates have been deposited in the Protein Data Bank under accession code xxxx.

### Homology modelling

The target (MDA5CARD1, RIGICARD1) and template (IPS-1 CARD) sequences were aligned and submitted to the program MODELLER [53] along with the IPS-1 CARD template structure. The model giving the lowest value of the MODELLER objective function was further refined using the loopmodel function in MODELLER and evaluated by calculating the DOPE (Discrete Optimized Protein Energy) score.

## List of abbreviations

CARD: caspase activation and recruitment domain. RIG-I: retinoic acid inducible gene-I. MDA5: melanoma differentiation-association gene 5.

## Authors' contributions

JAP designed the experiments, carried out all the experimental work and drafted the first manuscript. GLT & RER conceived the study and GLT analysed the structural data, produced the figures and developed the manuscript. All authors read and approved the final manuscript.
